# Silicon Nanowire Phototransistor Arrays for CMOS Image Sensor Applications

**DOI:** 10.3390/s23249824

**Published:** 2023-12-14

**Authors:** Hyunsung Jun, Johyeon Choi, Jinyoung Hwang

**Affiliations:** The School of Electronics and Information Engineering, Korea Aerospace University, Goyang-si 10540, Republic of Korea; gohighjun@kau.kr (H.J.); mandol0821@kau.kr (J.C.)

**Keywords:** bipolar junction transistor, CMOS image sensors, phototransistor, silicon nanowire

## Abstract

This paper introduces a new design of silicon nanowire (Si NW) phototransistor (PT) arrays conceived explicitly for improved CMOS image sensor performance, and comprehensive numerical investigations clarify the characteristics of the proposed devices. Each unit within this array architecture features a top-layer vertical Si NW optimized for the maximal absorption of incoming light across the visible spectrum. This absorbed light generates carriers, efficiently injected into the emitter–base junction of an underlying npn bipolar junction transistor (BJT). This process induces proficient amplification of the output collector current. By meticulously adjusting the diameters of the NWs, the PTs are tailored to exhibit distinct absorption characteristics, thus delineating the visible spectrum’s blue, green, and red regions. This specialization ensures enriched color fidelity, a sought-after trait in imaging devices. Notably, the synergetic combination of the Si NW and the BJT augments the electrical response under illumination, boasting a quantum efficiency exceeding 10. In addition, by refining parameters like the height of the NW and gradient doping depth, the proposed PTs deliver enhanced color purity and amplified output currents.

## 1. Introduction

In the contemporary digital age, the significance of color image sensing and processing is profoundly evident across various technological applications. Image sensors, the heart of this digital revolution, underpin the capabilities of devices ranging from digital cameras and smartphones to sophisticated medical imaging equipment and the burgeoning realm of autonomous vehicles. Over the past several decades, this field has witnessed unprecedented progress, marked by notable enhancements in resolution, dynamic range, and frame rate [1,2,3]. Nevertheless, despite these advancements, the technology has challenges and intrinsic limitations that necessitate further exploration and resolution.

The prevailing image sensor technologies on the market employ a color filter array (CFA) that incorporates organic dye color filters placed over silicon (Si) photodiode arrays [4]. However, the reduction in sensitivity in CFAs is inevitable with attempts to miniaturize pixel sizes, a trend driven by the pursuit of enhanced spatial resolution [5,6,7]. In addition, organic dye filters, essential to the CFA design, manifest vulnerabilities under elevated temperatures and exposure to ultraviolet radiation [3,8,9,10,11]. A further inherent limitation lies in the efficiency of color detection. The quantum efficiency of the total color detection is at most 1/3, attributed to the spatial partitioning required for RGB color separation [12]. An additional complication emerges during the fabrication process of these sensors. Constructing the three organic dye filters for the RGB patterns demands precise lithographic alignment for each color filter. This intricate process challenges the feasibility of producing multicolor imaging devices with complex array formats and extremely small-sized pixels [13,14].

Additionally, the conventional CMOS image sensors that utilize Si photodiodes typically exhibit a dynamic range of approximately 60–70 dB [15]. This figure is significantly lower than the human eye’s dynamic range, which exceeds 140 dB [2]. Such a constrained dynamic range impedes the sensor’s capability to accurately capture scenes characterized by high contrast or diverse lighting conditions, often resulting in the loss of detail in either highlights or shadows [16]. Furthermore, the quantum efficiency of the photodiode rarely surpasses unity, denoting a linear current response to incident light intensity [17]. Given this constrained conversion efficiency, additional circuit components are necessitated to bolster the device’s sensitivity under low-light conditions [18].

Several alternative methodologies have emerged in pursuit of solutions to the constraints imposed by organic color filters. These include photonic crystal color filters [19,20], metasurface color filters [21], color routers [22], and optical antennas [23,24]. Predominantly fabricated from inorganic semiconductors, these alternatives exhibit enhanced stability against thermal effects and ultraviolet radiation. Moreover, they are compatible with CMOS fabrication technology and exhibit enhanced performance even at the nanoscale pixel size. Among these, optical antennas constructed from arrays of vertical Si nanowires (NWs) with three different diameters and pixel sizes of 700 nm have exhibited quantum efficiency of total color detection that approaches unity [24]. The improved performance of these Si NW antennas can be attributed to their absorption cross-sectional area being considerably larger than their physical cross-sectional area [25,26,27,28]. This unique characteristic allows them to capture light from an extended surrounding area. Within this color detection scheme, each specific color channel effectively captures light from its designated spectral domain, ensuring minimal optical losses [29,30,31].

In this work, we adopted the Si NW antenna to design phototransistor (PT) arrays suitable for CMOS image sensor applications. Each PT features a Si NW atop a vertically structured bipolar junction transistor (BJT), wherein the bottom of the Si NW establishes a connection to the base of the BJT. The design incorporates three variants of PTs, each differentiated by the diameter of the Si NW to detect blue, green, and red colors, selectively. The arrangement of these PTs emulates the Si NW antenna’s configuration to capitalize on its superior light absorption efficacy and color discrimination capabilities. Moreover, the BJT structure is meticulously optimized to efficiently inject photogenerated carriers from the Si NW into the BJT’s base. This design strategy culminates in a pronounced amplification of the output current signals, subsequently elevating the quantum efficiency in total color detection. The characteristics of the proposed devices were unveiled through numerical simulations, with 3D finite-difference time-domain (FDTD) simulations employed to scrutinize their optical attributes and 3-D technology computer-aided design (TCAD) simulations to assess their electrical characteristics.

## 2. Results and Discussion

**Figure 1:**
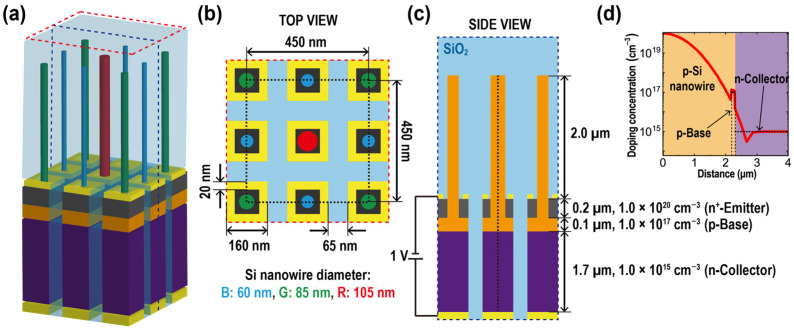
(**a**) A 3-D perspective of the Si NW PT array where PTs are periodically arranged. (**b**) Top view of the PT array, corresponding to the region delineated by the red dashed lines in (**a**). Colors have been added to indicate absorption according to the wavelength, which varies with diameter: 60nm absorbs blue (440 nm), 85 nm absorbs green (540 nm), and 105 nm represents red (610 nm). (**c**) Side view of the PT array, demarcated by blue dashed lines in (**a**). Colors have been assigned to represent the emitter, base, and collector according to doping concentration. (**d**) The gradient profile of the doping concentration in the Si NW is plotted along the black dashed line in (**c**).

Figure 1a describes a periodic array of Si NW PTs designed for CMOS image sensor applications. In this configuration, individual devices, labeled as B-PT, G-PT, and R-PT, absorb specific regions of the visible light spectrum, namely, blue, green, or red, and the distinct colorations of their NWs differentiate them. The Si NWs at the top of each device predominantly absorb incident light from above, offering the capability to modulate the peak absorption wavelength through precise adjustments to the NW diameter. Figure 1b illustrates the top view of the periodic PT array, referring to the region outlined by red dashed lines in Figure 1a. Delineated by black dashed boundaries, each square unit cell spans a side length of 450 nm and comprises four PTs: two designed for blue light absorption, one for green, and another for red. This configuration is optimized to equilibrate the absorption cross-sectional area of NWs because the NWs absorbing blue light have a smaller cross-sectional area than those for green and red. The unit cell stands as a pixel in the context of CMOS image sensors. The 450 nm pitch of this design is approximately 100 nm less than the pixel pitch in state-of-the-art commercial CMOS image sensors. The diameters of the Si NWs absorbing blue, green, and red light, designated as B-NW, G-NW, and R-NW, are 60, 85, and 105 nm, respectively. The determination of each NW’s diameter is guided by the formula d ≈ 0.67 × λ/*n*_si_, where d represents the diameter of an NW, λ is the wavelength of the incident light, and *n*_si_ is the refractive index of crystalline Si [24]. This equation asserts that for NWs of a specified diameter, light with a particular wavelength resonates in HE_11_ mode within the NW, leading to pronounced light absorption at that specific wavelength. Accordingly, by utilizing this equation, the diameters of the NWs are determined, aligning with achieving peak absorption at the wavelengths corresponding to three peaks of the CIE 1964 XYZ color-matching functions, namely, 440 nm, 550 nm, and 600 nm. The oxide-filled gap between PTs of 65 nm effectively eliminates the potential for electrical crosstalk between neighboring contacts.

Figure 1c showcases the side view of the periodic PT array, as restricted by blue dashed lines in Figure 1a. It reveals the device structure of the PTs, with a Si NW integrated atop an underlying npn BJT. The BJT features an uppermost n-type emitter with a thickness of 0.2 μm, possessing a high doping concentration of 10^20^ cm^−3^. Beneath the emitter layer lies a 0.1 μm thick p-type base with a doping concentration of 10^17^ cm^−3^. The 1.7 μm thick n-type bottom-most collector has a doping concentration of 10^15^ cm^−3^. A p-type 2 μm long Si NW penetrates the emitter layer and connects to the base. The Si NW exhibits a Gaussian doping concentration profile decreasing from top to bottom. This nonuniform doping profile augments the injection efficiency of the photogenerated carriers from the NW to the base by establishing an induced electric field, producing a force on the electrons toward the base. Figure 1d presents the gradient doping profile, plotted along the black dashed line in Figure 1c, which is at the center of the device from the top of the Si NW to the bottom of the collector. While the base remains without external contact, the device operates with a 1 V bias at the collector, with the top emitter grounded.

**Figure 2:**
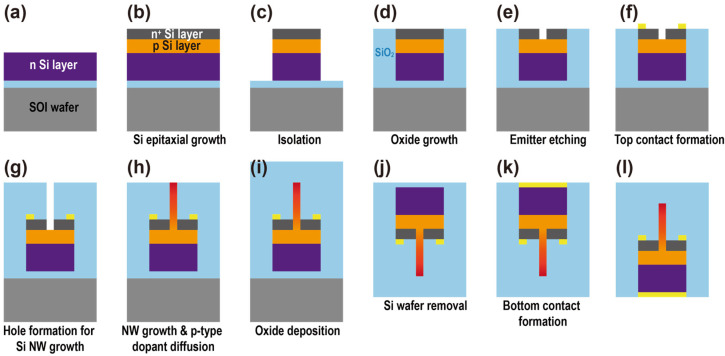
Sequential steps of device fabrication utilizing traditional Si processing techniques: (**a**) initial SOI wafer configuration, (**b**) Si epitaxial growth forming n^+^ and p layers, (**c**) isolation of the device area, (**d**) growth of SiO_2_ layer, (**e**) emitter region etching, (**f**) formation of top electrical contact, (**g**) creation of holes for subsequent Si NW growth, (**h**) NW growth accompanied by p-type dopant diffusion, (**i**) deposition of an oxide layer, (**j**) removal of the Si wafer substrate, (**k**) formation of the bottom electrical contact, and (**l**) final device architecture with contacts.

Figure 2 outlines the proposed device fabrication process employing standard Si fabrication technologies. The selection of fabrication methods is based on considerations of practical implementation feasibility. The process commences with a silicon-on-insulator (SOI) wafer (a), followed by the successive deposition of three epitaxial layers: n^+^-emitter, p-base, and n-collector (b). After this, each BJT device undergoes isolation through mask creation and etching (c). Notably, a deep trench between devices, with a width of 65 nm and a depth of 2 μm featuring an aspect ratio of 31:1, is achieved through deep Si etching techniques, such as deep reactive ion etching [32]. An oxide layer encapsulates the BJTs after isolation through thermal oxide growth (d). The central portion of the emitter is removed via electron beam (e-beam) lithography for mask creation, followed by Si etching, revealing the base surface intended for Si NW growth (e). An emitter contact is defined atop the emitter layer (f), with the linewidth of the contact set at 20 nm, achievable through e-beam lithography and a subsequent lift-off process [33]. The process continues with the deposition of an oxide layer through physical vapor deposition, and an aperture is created using e-beam lithography, followed by the oxide etching process to facilitate the upward growth of the Si NW (g). The 2 μm long Si NWs are vertically grown from the base surface through the vapor–liquid–solid growth process [34], and they are subjected to p-type doping via thermal diffusion (h). An additional oxide layer is uniformly deposited across the entire device to protect the underlying devices from potential damage during the subsequent fabrication steps (i). The bottom Si substrate is removed through chemical-mechanical polishing (j). After etching away the buried oxide layer below the device area using e-beam lithography and a subsequent oxide etching process, a bottom collector contact is formed on the Si surface through a lift-off process (k,l). The detailed fabrication steps are illustrated in Supplementary Information Figure S1.

**Figure 3:**
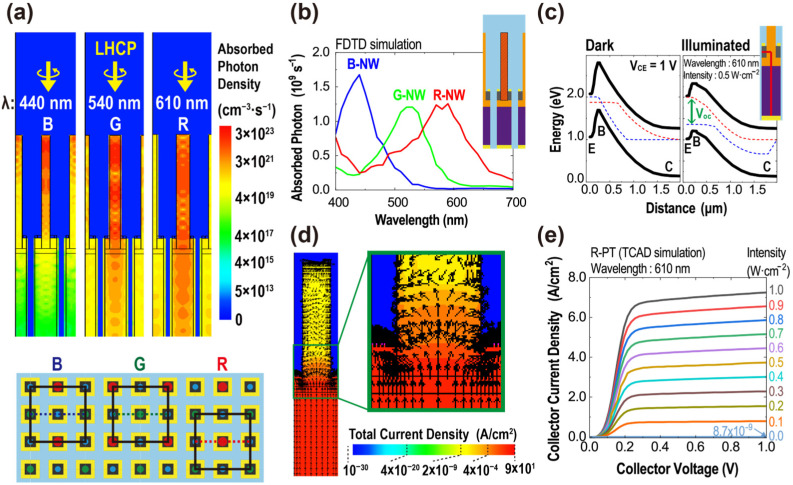
(**a**) Spatial photon absorption profiles for three distinct incident wavelengths: 440 nm (B), 540 nm (G), and 610 nm (R). Dashed lines of corresponding colors on the top view of the PT array below highlight the regions from which these profiles are extracted, with black lines demarcating the periodic boundaries of the simulation domain. (**b**) The number of absorbed photons as a function of incident light wavelength within the NW region, depicted as the diagonally hatched area in the inset figure, as a function of wavelength. (**c**) Energy band diagrams of the R-PT at V_CE_ = 1 V under two conditions: dark and illuminated, traced along the red line displayed in the inset. The red dotted line represents the Quasi-Fermi level of electrons, and the blue dotted line represents the Quasi-Fermi level of holes. (**d**) Total current density distribution of the R-PT obtained at the illuminated condition described in (**c**). Superimposed black arrows indicate the direction of the current flow. (**e**) The output characteristics of the PTs.

FDTD calculation provides the light absorption characteristics of the periodic PT array in the visible range of the optical spectrum spanning from 400 to 700 nm. The light source utilized in the optical simulation is a normally incident left-handed circularly polarized plane wave. Figure 3a depicts absorbed photon density distributions at three distinct incident wavelengths: 440 nm (B), 540 nm (G), and 610 nm (R). These wavelengths are associated with the HE_11_ resonance mode of Si NWs with respective diameters of 60 nm, 85 nm, and 105 nm. The distribution data are derived from the locations highlighted by color-matched dashed lines on the top view of the PT array, with black lines defining the periodic boundaries of the simulation domain. Light absorption predominantly occurs within the NWs. In addition, at each resonance wavelength, photon absorption is pronounced in the corresponding NW, while the other NWs exhibit substantially reduced absorption. Figure 3b plots the number of absorbed photons within the NW region, represented as the diagonally hatched area in the inset figure, as a function of wavelength. Absorption spectra from the NWs of different diameters exhibit peaks at 440, 540, and 610 nm, each corresponding to distinct light absorption in the blue, green, and red spectral regions. This observation aligns with prior research suggesting that the HE_11_ resonance mode of Si NW offers adequate spectral selectivity for color discrimination in image sensors [24].

TCAD simulations were employed to assess the electrical characteristics of the PTs. Figure 3c portrays the energy band diagrams of the PT associated with the red-light-absorbing NW (R-PT), traced along the red line illustrated in the inset, encompassing the emitter, base, and collector regions. Under the dark condition, with a potential difference of 1 V between the emitter and collector (V_CE_ = 1 V), the PT remains in cutoff mode, resulting in negligible current flow from the collector to the emitter. However, upon illumination with a light of 610 nm wavelength at an intensity of 0.5 W/cm^2^, the photogenerated carriers are injected from the NW to the base, inducing the separation of the quasi-Fermi levels in the base. Owing to the open-circuit voltage (V_OC_) established in the base region, the emitter–base junction shifts to a forward-bias state, prompting the device to enter forward active mode. The transition leads to a notable current flowing from the collector to the emitter, as visualized in the total current distribution in Figure 3d. Figure 3e shows the output characteristics of the PTs, displaying the conventional I_C_–V_CE_ curves of an npn BJT operating in the common emitter configuration. The output currents are approximately proportional to the input parameter, specifically, the intensity of incident light.

**Figure 4:**
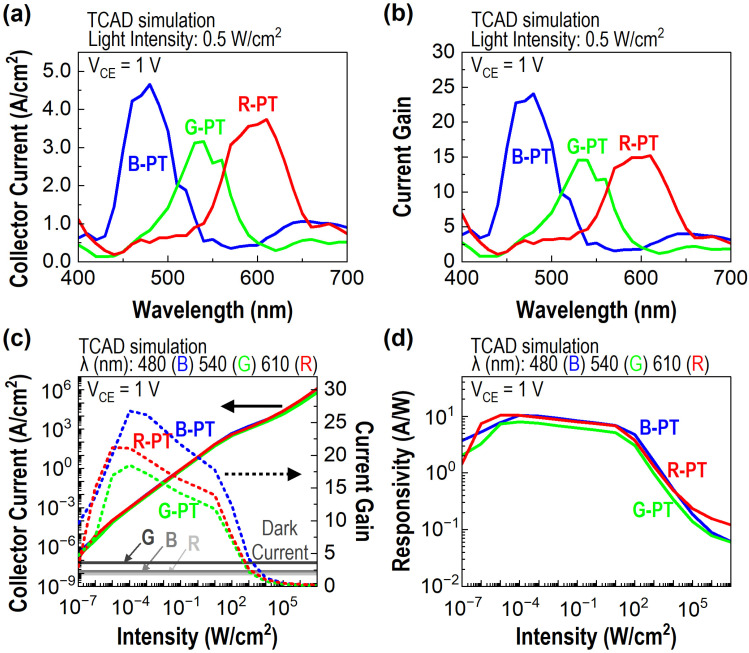
(**a**) Collector current density of three PTs at V_CE_ = 1 V, plotted against the wavelength of incident light at an intensity of 0.5 W/cm^2^. (**b**) The current gain for three PTs concerning the wavelength of incident light. (**c**) The collector current of three PTs at V_CE_ = 1 V as a function of light intensity at the peak wavelengths of their output current spectra: 480, 540, and 610 nm. Dashed lines in matching colors represent corresponding current gains. Three gray curves depict the dark currents at V_CE_ = 1 V. The black solid line arrow represents the collector current (A/cm^2^), and the black dotted line arrow indicates the current amplification factor. (**d**) Responsivity for three PTs estimated from the collector current data in (**c**).

Figure 4a provides the collector current density for three PTs—B-PT, G-PT, and R-PT—at V_CE_ = 1 V for the wavelength of incident light under an illumination intensity of 0.5 W/cm^2^ on the PT array. The output current spectrum of each PT closely resembles the photon absorption spectrum of the NW incorporated into the corresponding PT, as depicted in Figure 3b. The similarity underscores the prominent color discrimination attributes of the PTs. Additionally, the results suggest that the injection of photon-generated carriers from the NW predominantly governs the current flow between the emitter and collector. Notably, in the case of the B-PT, the peak of the output current spectrum exhibits a peak at a longer wavelength than the peak observed in the NW photon absorption spectrum. As the wavelength increases, photon absorption near the emitter–base junction intensifies. This phenomenon is favorable for the injection of carriers into the junction. For further clarity, although the NW absorbs more photons at 440 nm, the influx into the emitter–base junction at 480 nm surpasses the influx at 440 nm. It leads to an augmented forward bias current at this junction, culminating in the maximum output collector current at a wavelength of 480 nm. Figure 4b elucidates the current gain of the three PTs, quantified as the ratio between the electron flux traversing the collector contact and the photon flux incident on the device. The PTs consistently exhibit gain values exceeding 10 within their operational wavelength spectrum. Such results suggest that the integrated bottom BJTs amplify the incoming light signal efficaciously.

Figure 4c delineates the collector current for the B-PT, G-PT, and R-PT as a function of light intensity, corresponding to their respective peak wavelengths: 480, 540, and 610 nm. The devices produce an output current beyond the dark current at a light intensity as low as 10^−7^ W/cm^2^, implying their potential utility as sensors capable of functioning under remarkably low light intensities. The current gain spectra illustrated in Figure 4c demonstrate that the optimal operational intensity for these devices spans from 10^−6^ to 10^2^ W/cm^2^. Within this range, the current gain for the three PTs consistently exceeds a value of five at their designated peak wavelengths. Beyond an intensity of 10^2^ W/cm^2^, the current gain of the PTs considerably decreases. This attenuation can be attributed to high injection and series resistance, which impede the injection efficiency of the PTs. Furthermore, Figure 4d illustrates that an evaluation of responsivity, derived from the collector current depicted in Figure 4c, yields values ranging from 3 to 10 A/W within the optimal operational intensity range. This performance metric is approximately a tenfold improvement over that of conventional Si photodetectors employed in CMOS image sensors.

**Figure 5:**
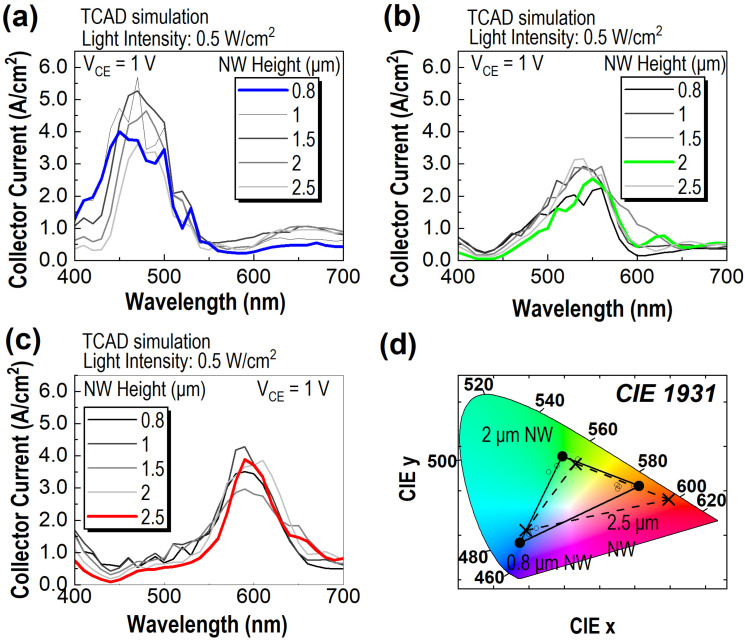
(**a**–**c**) Collector current spectra for B-PT, G-PT, and R-PT at V_CE_ = 1 V across different NW heights under a light intensity of 0.5 W/cm^2^. (**d**) The CIE 1931 chromaticity diagram. Circles demarcated by black boundaries on the diagram define data points derived from the collector current spectra shown in (**a**–**c**). Black crosses represent data points for a CFA.

To further improve device performance, specific physical parameters of the PTs are adjusted, including the NW height and the gradient doping depth of the NWs. Firstly, the NW height is varied to augment the chromaticity of the PTs. Figure 5a displays the collector current spectrum for B-PT at V_CE_ = 1 V across varied NW heights under a light intensity of 0.5 W/cm^2^. A noticeable blueshift occurs in the spectrum’s peak as the NW height decreases. This observation correlates with the discrepancy between the peak positions of the collector current spectrum for B-PT (as shown in Figure 4a) and the photon absorption spectrum of the NW in B-PT (presented in Figure 3b). As the NW height decreases, the top becomes more proximate to the emitter–base junction, increasing the chance for photons absorbed atop the NW to reach the emitter–base junction. It ensures that most photons absorbed in shorter NWs can be directed into the emitter–base junction, leading the peak of the output current spectrum to coincide with the peak of the NW’s photon absorption spectrum. Figure 5b,c give the output current spectrum for the G-PT and R-PT at V_CE_ = 1 V for various NW heights under the light intensity of 0.5 W/cm^2^. The spectra of both PTs manifest a blueshift as the NW height diminishes, albeit less pronounced compared to that of B-PT.

Figure 5d shows the CIE chromaticity diagram, elucidating the color purity of the PTs. The circles demarcated with black boundaries in the diagram are derived from the collector current spectra showcased in Figure 5a–c. For optimized chromaticity, NW heights of 0.8, 2, and 2.5 μm are selected for the B-PT, G-PT, and R-PT, respectively. The spectra associated with these height choices appear with their absorption colors in Figure 5a–c. For comparison, the chromaticity data from a conventional CFA are also included in the CIE diagram [4]. This comparative analysis reveals that B-PT and G-PT manifest enhanced color purity relative to the CFA. Conversely, R-PT would benefit from further design refinements to transcend the efficacy of existing technologies.

Furthermore, the gradient doping depth of the NWs incorporated in the PTs is modified to control the magnitude of the collector current. The depth is equivalent to the position at which the doping concentration of the p-type Gaussian doping profile within the NWs reaches 5 × 10^16^ cm^−3^. Assuming that the gradient doping originates from thermal diffusion, the doping concentration at the top of the NW remains consistent at 10^20^ cm^−3^. While maintaining this top concentration, a reduction in gradient doping depth approximates a decrease in the variance of the Gaussian distribution, resulting in a more sharply varying doping concentration. The increased doping variation rate amplifies the induced electric field within the NW, which, in turn, accelerates the injection of photogenerated carriers from the NW to the emitter–base junction. Consequently, the collector current amplifies as the gradient doping depth of the NW decreases.

**Figure 6:**
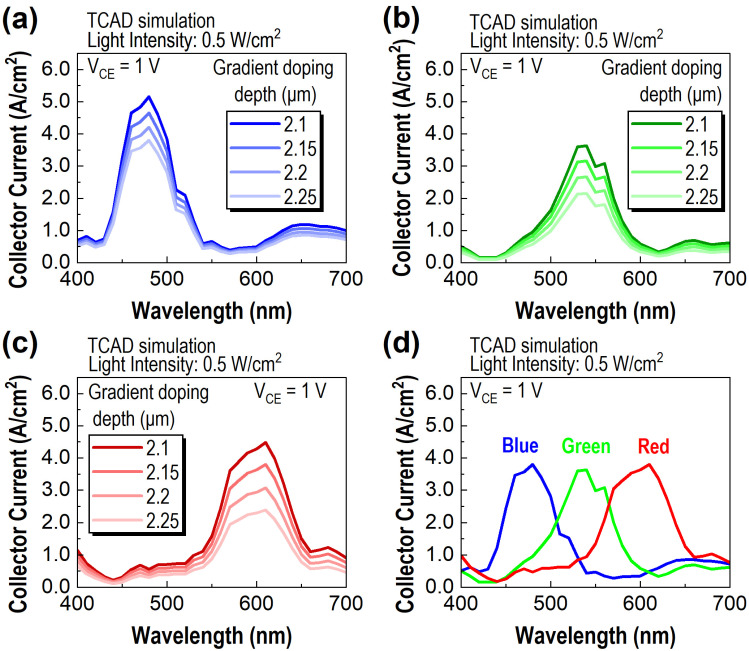
(**a**–**c**) Collector current spectra at V_CE_ = 1 V for B-PT, G-PT, and R-PT, respectively, showcasing variations with different gradient doping depths of the NWs integrated into the PTs, all under a light intensity of 0.5 W/cm^2^. (**d**) Collector current spectra for B-PT, G-PT, and R-PT correspond to gradient doping depths of 2.25 μm, 2.2 μm, and 2.15 μm, respectively, which exhibit roughly equivalent peak current intensities.

Data obtained from the B-PT, G-PT, and R-PT, calculated at V_CE_ = 1 V and a light intensity of 0.5 W/cm^2^, follow the trend described in Figure 6a–c, respectively. By utilizing the results, three PTs were designed to yield comparable output currents at the peak wavelength of their respective collector current spectra. As represented in Figure 6d, B-PT, G-PT, and R-PT—with gradient doping depths of 2.25, 2.2, and 2.15 μm, respectively—deliver collector currents of 3.80, 3.63, and 3.80 A/cm^2^ at their peak wavelengths of 480, 540, and 610 nm.

## 3. Conclusions

In conclusion, we developed a novel design of Si NW PT arrays specifically tailored for CMOS image sensors. The performance characteristics of the devices were validated through detailed numerical simulations. Each PT comprises a top Si NW and a bottom npn BJT. The top Si NW efficiently absorbs most of the incoming light, and the resulting photogenerated carriers are subsequently injected into the emitter–base junction of the bottom BJT, facilitating a notable amplification of the output collector current. By carefully manipulating the NW diameters, the PTs manifest distinct absorption characteristics for the visible spectrum’s blue, green, and red regions. Moreover, the PTs present an enhanced electrical response upon illumination, achieved through the proficient current amplification capabilities of the BJT. Through optimizing device parameters, such as NW height and gradient doping depth, these PTs ensure improved color purity and yield elevated output currents, outperforming conventional Si photodetectors in image sensors.

## Data Availability

The data presented in this study are available on request from the corresponding author.

## Supplementary Materials

The following supporting information can be downloaded at: https://www.mdpi.com/article/10.3390/s23249824/s1, Figure S1: Sequential steps of device fabrication utilizing traditional Si processing techniques.
