# Supplementary material for: Silicon Nanowire Phototransistor Arrays for CMOS Image Sensor Applications
Source: Sensors (Basel). 2023 Dec 14;23(24):9824. doi: 10.3390/s23249824 (PMC10748017; doi:10.3390/s23249824)
Supplement: Supplementary file 1 [file sensors-23-09824-s001.zip › sensors-2754043-supplementary.pdf]

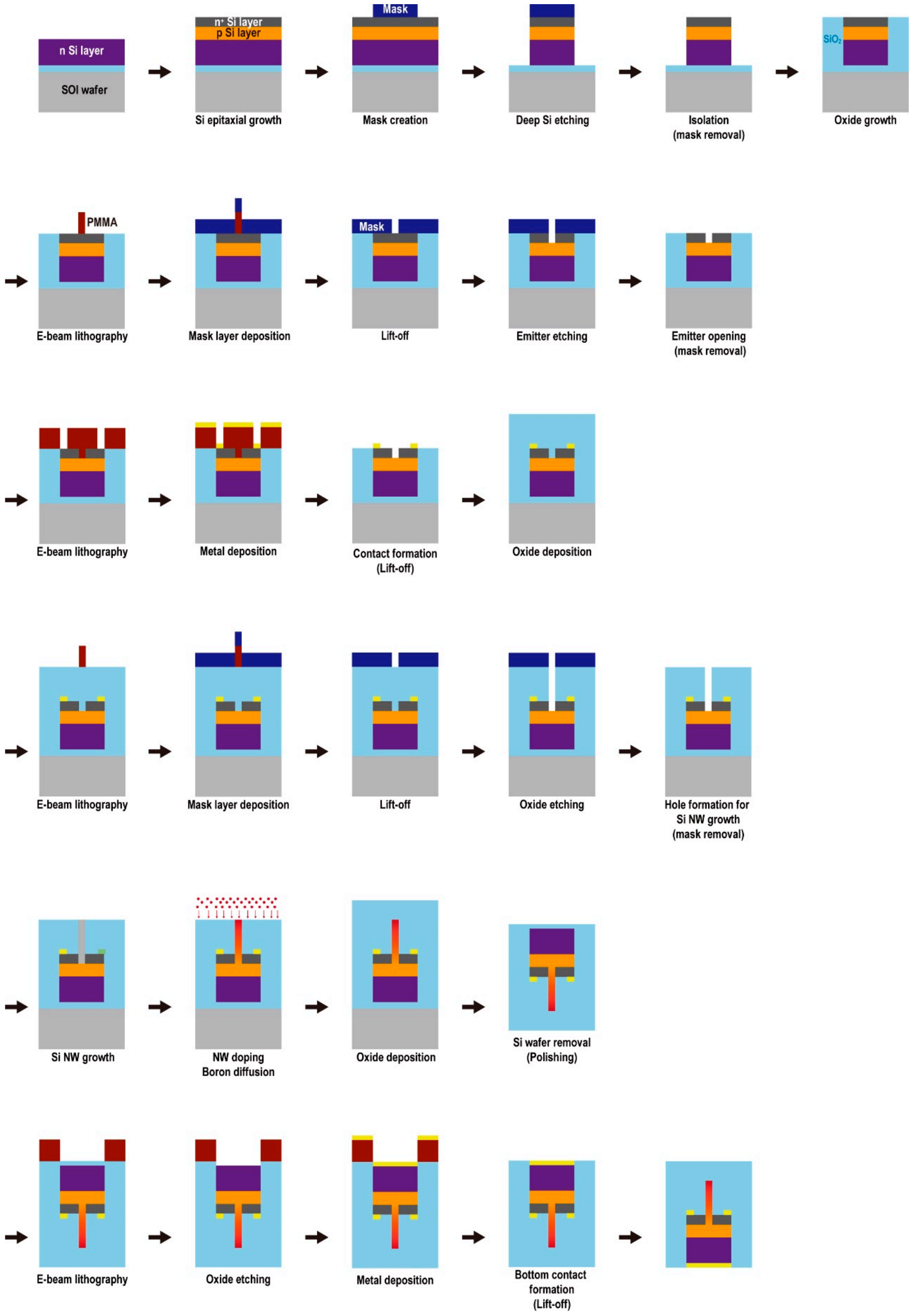

Figure S1. Sequential steps of device fabrication utilizing traditional Si processing techniques.
